# Chronic Stress Alters Spatial Representation and Bursting Patterns of Place Cells in Behaving Mice

**DOI:** 10.1038/srep16235

**Published:** 2015-11-09

**Authors:** Mijeong Park, Chong-Hyun Kim, Seonmi Jo, Eun Joo Kim, Hyewhon Rhim, C. Justin Lee, Jeansok J. Kim, Jeiwon Cho

**Affiliations:** 1 Center for Neuroscience, Korea Institute of Science and Technology, 5 Hwarang-ro 14-gil, Seongbuk-gu, Seoul 136-791, Korea; 2 Center for Functional Connectomics, Korea Institute of Science and Technology, 5 Hwarang-ro 14-gil, Seongbuk-gu, Seoul 02792, Korea; 3 Neuroscience Program, Korea University of Science & Technology, 217 Gajeong-ro, Daejeon 34113, Korea; 4 Department of Psychology, University of Washington, 3921 West Stevens Way Northeast, Seattle, WA 98195-1525, USA

## Abstract

Chronic uncontrollable stress has been shown to produce various physiological alterations and impair mnemonic functions in the rodent hippocampus. Impacts on neuronal activities, however, have not been well investigated. The present study examined dorsal CA1 place cells to elucidate the computational changes associated with chronic stress effects on cognitive behaviors. After administering chronic restraint stress (CRS; 6 hours/day for ≥21 consecutive days) to adult male mice, several hippocampal characteristics were examined; i.e., spatial learning, *in vitro* synaptic plasticity, *in vivo* place cell recording, and western blot analysis to determine protein levels related to learning and memory. Behaviorally, CRS significantly impeded spatial learning but enhanced non-spatial cue learning on the Morris water maze. Physiologically, CRS reduced long-term potentiation (LTP) of Schaffer collateral/commisural-CA1 pathway, phospho-αCaMKII (alpha Ca2^+^/calmodulin-dependent protein kinase II) level in the hippocampus, and stability of spatial representation and the mean firing rates (FRs) of place cells. Moreover, the local cue-dependency of place fields was increased, and the intra-burst interval (IntraBI) between consecutive spikes within a burst was prolonged following CRS. These results extend the previous findings of stress impairing LTP and spatial learning to CRS modifying physical properties of spiking in place cells that contribute to changes in navigation and synaptic plasticity.

The hippocampus is crucial for the formation of long-term declarative (or explicit) memory in humans and spatial (or relational) memory in rodents^1^,^2^,^3^,^4^. It is also implicated in inhibiting the hypothalamus-pituitary-adrenal (HPA) neuroendocrine response to stress^5^,^6^,^7^. As receptors for corticosteroids (cortisol in human and corticosterone in rodent) are concentrated in the hippocampus, a number of human and animal studies indicate that hippocampus-based learning and memory functions are susceptible to uncontrollable stress. In humans, impairments in verbal recall tasks have been observed in individuals diagnosed with posttraumatic stress disorders (PTSD)^8^ and Cushing’s disease characterized by hypercortisolaemia^9^. Subnormal memory performances have also been demonstrated in healthy subjects who received high doses of cortisol and/or were exposed to audiogenic stress^10^,^11^. In rodents, inescapable/unpredictable stress produces impairments in various spatial memory tasks^12^,^13^,^14^. Interestingly, stress that impedes hippocampal-based learning has been found to enhance competing hippocampal-independent learning in rats^15^ and humans^16^. Thus, the detrimental mnemonic effects of stress seem to be particular to hippocampal-dependent tasks.

As the magnitude of stress increases, a number of transient-to-lasting physiological changes that can influence mnemonic functioning have been identified in the hippocampus^17^. Intense acute stress (e.g., restraint + tailshocks) in rats has been shown to weaken the induction of LTP, a putative synaptic model of information storage, in the hippocampus for up to 48 hours^18^. With chronic stress (e.g., recurrent restraint across days), morphological changes (e.g., dendritic retraction), suppression of adult neurogenesis, and neuronal endangerment have been observed in the hippocampus^19^,^20^. While these stress-associated changes have been extensively investigated, much less is known about the effects of stress on neuronal activities in behaving animals. The pyramidal neurons in rodent hippocampus display characteristic burst activities when the animal enters a specific location of a familiarized environment^21^,^22^,^23^. The bursting is an electrophysiological signature of pyramidal neurons and appears to represent an important form of information coding in the hippocampus^24^. Due to the location-specific firing property, these “place cells” are thought to play vital functions in navigation-based learning and memory^2^,^21^. Accordingly, place cells can provide valuable information as to how stress influences the hippocampus at the neural computational level, and thereby fill the gaps between cellular, morphological, and cognitive changes associated with stress. An earlier study found that following an acute audiogenic stress (2 h) experience, rats exhibited decreases in spatial correlation and stable firing pattern in their place cells^25^. Similarly, 30 min of acute photic stress exposure significantly decreased the firing rates of CA1 and CA3 place cells^26^. The extent of chronic stress influences on place cells has just begun to be studied. A very recent study found that restraining mice 2 h/d for 5 consecutive days (but not 10 consecutive days due to adaptation) caused significant decreases in firing rates and field sizes of place cells^27^. However, this study focused exclusively on the place cell properties; whether the same stress affected other hippocampal functions, such as spatial learning and synaptic plasticity, are unknown. Thus, we investigated several neurophysiological effects of CRS in mice (Fig. S1), and report that the same stress that impaired spatial learning and LTP also decreased the stability of place fields, the mean FR and the phospho-αCaMKII level, altered the bursting pattern of place cells, and shifted the place fields’ dependency from spatial cues to local cues.

## Results

### General effects of CRS

During CRS procedure, stressed mice showed significant decrease in body weights compared to control mice (one-way repeated measures ANOVA: *F*_(1,34)_ = 5.37, *P* = 0.02, main effect of group; *F*_(5,186)_ = 2.48, *P* = 0.01, group x day interaction) (Fig. S2a). The plasma corticosterone (CORT) levels, measured using additional groups of animals (n = 3 mice/measurement time/group) (Fig. S2b), further indicated the continuing aversiveness of CRS in mice (day 1, *z* = −2.3, *P* = 0.02; day 10, *z* = −2.3, *P* = 0.02; day 21, *z* = −2.32, *P* = 0.02).

CRS-induced morphological changes were also observed in both CA1 (control: 14 neurons, 3.5 + 0.64 neurons/mouse; stress: 13 neurons, 3.25 + 0.62 neurons/mouse) and CA3 (control: 18 neurons, 4.5 + 0.5 neurons/mouse; stress: 14 neurons, 3.5 + 0.97 neurons/mouse) fields following Golgi-staining (Fig. S2c). The spine numbers per matched 50 μm segments were reliably lower in stressed group compared to control group in both CA1 (*z* = −3.7, *P* < 0.01) and CA3 (*z* = −2.36, *P* = 0.01) regions (Fig. S2d). These results indicated that CRS paradigm employed in the present study was effective in mice, consistent with previous studies^28^,^29^.

### CRS reduced hippocampal LTP and phospho-αCaMKII protein level

**Figure 1:**
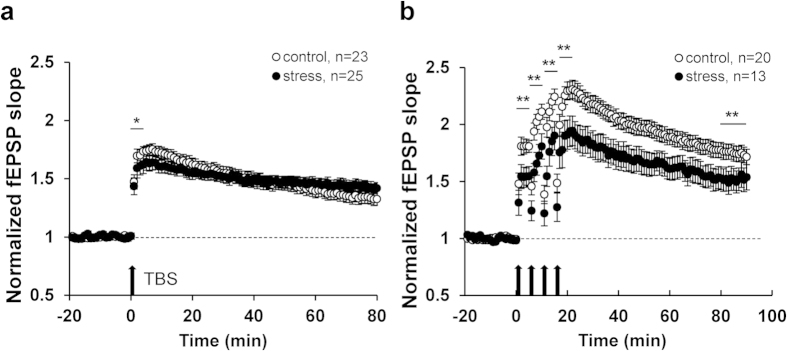
Properties of basic synaptic transmission and synaptic plasticity at CA1 hippocampal synapse *in vitro*. (**a**) Single TBS induced LTP. (**b**) Four TBS induced LTP. All values are presented as the mean + SEM (Unpaired two-tailed *t*-test, *P < 0.5, **P < 0.01).

After 21 day exposure to CRS, *in vitro* synaptic plasticity was assessed in the Schaffer collateral/commisural-CA1 pathway. The stressed mice showed significantly reduced short-term potentiation (the 1^st^ 5 min after TBS) (*t*_(238)_ = 2.53, *P* = 0.01) (Fig. 1a). Under four TBS (required for new protein synthesis^30^), CRS reduced short-term potentiation immediately after each of four TBS (at 0 ~ 5 min: *t*_(162)_ = 4.43, *P* < 0.01; at 6−10 min: *t*_(162)_ = 5.04, *P* < 0.01; at 11–15 min: *t*_(162)_ = 4.33, *P* < 0.01 and at 16–20 min: *t*_(162)_ = 3.72, *P* < 0.01) and also declined LTP (at 80 ~ 90 min after the 1^st^ TBS application) (*t*_(307)_ = 6.24, *P* < 0.01) (Fig. 1b). However CRS did not affect the intrinsic properties (i.e., membrane potential, membrane capacitance and # of action potential) of hippocampal CA1 pyramidal neurons and basal synaptic transmission (i.e., Input-Output curve; I/O curve and paired-pulse facilitate ratio; PPF ratio) from CA3 to CA1 synapses (Fig. S3a–e). Taken together, the results indicate that CRS does reduce the spine number and LTP of CA1 pyramidal neurons, while affecting little the basal properties of synaptic transmission of remaining synapses and the intrinsic excitability of CA1 cells.

The western blot analysis was conducted to access the effect of CRS on the protein levels of αCaMKII and phospho-αCaMKII in the CA1 area. Overall, the stressed mice showed a trend of increased αCaMKII protein level (*t*_(10)_ = −2.1, *P* = 0.054) but a significantly decreased phospho-αCaMKII protein level (*t*_(10)_ = 2.96, *P* = 0.02) compared to the control mice (Fig. S4a,b).

### CRS impaired hippocampal dependent spatial learning and memory in hidden platform water maze task

**Figure 2:**
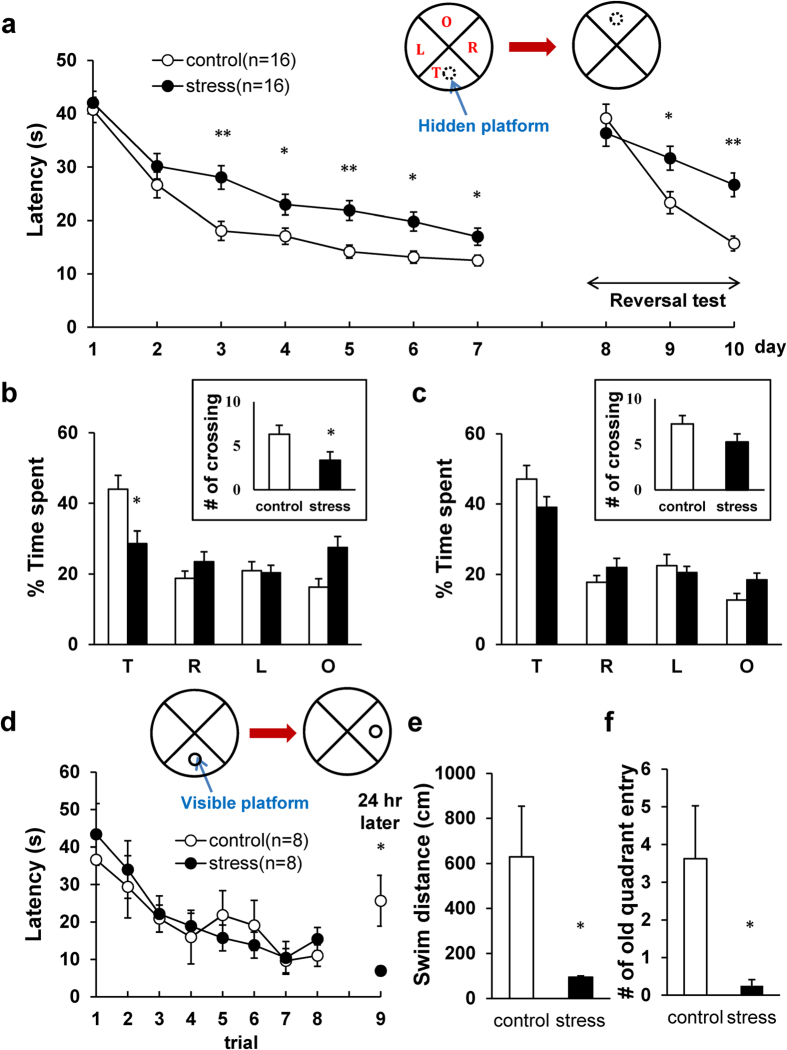
CRS effects on water maze tasks. (**a–c**) Hidden platform water maze task. (**a**) Latency to find hidden platform during acquisition and reversal learning. (**b,c**) % time spent in 4 quadrants during probe tests on days 3 and 8; *(Inset)* Platform crossing number during probe tests. T, target; R, adjacent right; L, adjacent left; O, opposite quadrant. (**d–f**) Visible platform task. (**d**) Latency to reach visible platform during training and retention test 24 hours later. (**e**) Distance to reach visible platform during the retention test. (**f**) The number of old quadrant entry where the platform was located during training. All values are presented as the mean + SEM (One-way repeated ANOVA, Unpaired two-tailed *t*-test, *P < 0.05, **P < 0.01).

The Morris water maze was used to assess the effects that CRS exerts on hippocampal-dependent spatial learning and memory. During the hidden platform training, stressed and control mice showed a significant group difference in acquisition (one-way repeated measures ANOVA: *F*_(1.129)_ = 22.39, *P* < 0.01, main effect of group) but there was no group x day interaction (*F*_(5.774)_ = 1.10, *P* = 0.3) (Fig. 2a). Although the swim speed was initially slower in stressed mice (one-way repeated measures ANOVA: *F*_(1,128)_ = 44.57, *P* < 0.01, main effect of group; *F*_(7,1152)_ = 3.74, *P* < 0.01, group x day interaction) (Fig. S5a), it did not appear to be due to any motor or sensory deficits caused by CRS since differential swim speed did not affect the performance during the 1^st^ and 2^nd^ training day. Consistent with the acquisition data, the probe tests (Fig. 2b,c) indicated that stressed mice spent less time swimming in the quadrant where a hidden platform was placed during training than control mice did (the 1^st^ probe test: *t*_(32)_ = 2.3, *P* = 0.02 in the target quadrant; the 2^nd^ probe test: *t*_(32)_ = 1.64, *P* = 0.05 in the target quadrant). The stressed mice also exhibited less platform crossing (1^st^ probe test: *t*_(32)_ = 2.3, *P* = 0.005); however the group difference was not statistically reliable on the 2^nd^ probe test (*t*_(32)_ = 1.6, *P* = 0.11).

In addition, after 7 days of the hidden platform testing, the platform was moved to the opposite quadrant to assess the animal’s ability to learn new spatial location as reversal learning for 3 days. The stressed mice were impaired in finding a new hidden platform location (one-way repeated measures ANOVA: *F*_(1,129)_ = 8.53, *P* = 0.004, main effect of group; *F*_(2,258)_ = 5.67, *P* = 0.004, group x day interaction). The reversal probe test revealed that stressed mice spent more time swimming in the acquisition target quadrant (*t*_(32)_ = −2.08, *P* = 0.04) (Fig. S5b,c), suggesting that CRS impaired cognitive flexibility^31^,^32^.

### CRS strengthened hippocampal independent Stimulus-Response (S-R) task in visible platform water maze task

To determine whether CRS effects on water maze was specific to a spatial task, different cohort of stressed and control animals underwent water maze training using a visible platform, an S-R task which does not require the hippocampus^33^. On this task, where both spatial and discrete cues are available, there was no group difference in the latency to find the platform during the 8 training trials (one-way repeated measures ANOVA: *F*_(1,14)_ = 0.1, *P* = 0.75, main effect of group; *F*_(7,98)_ = 0.38, *P* = 9.08, group x trial interaction) (Fig. 2d). However, when the visible platform was moved to the adjacent right quadrant 24 hours later, stressed mice showed a significantly shorter latency (*t*_(7)_ = 2.7, *P* = 0.02) and swim distance (*t*_(7)_ = 2.3, *P* = 0.04) to find the new platform than control mice (Fig. 2e). In contrast, control mice showed higher number of old platform location entry than stressed mice (*t*_(7)_ = 2.39, *P* = 0.04) (Fig. 2f). These differences did not appear to be due to motoric effects, because there was no group difference in swim speed (*t*_(14)_ = −0.06, *P* = 0.54) (Fig. S5d).This finding indicates that CRS exerts contrasting influences on hippocampal-dependent spatial *vs*. hippocampal-independent S-R tasks.

### CRS altered place cell properties

**Figure 3:**
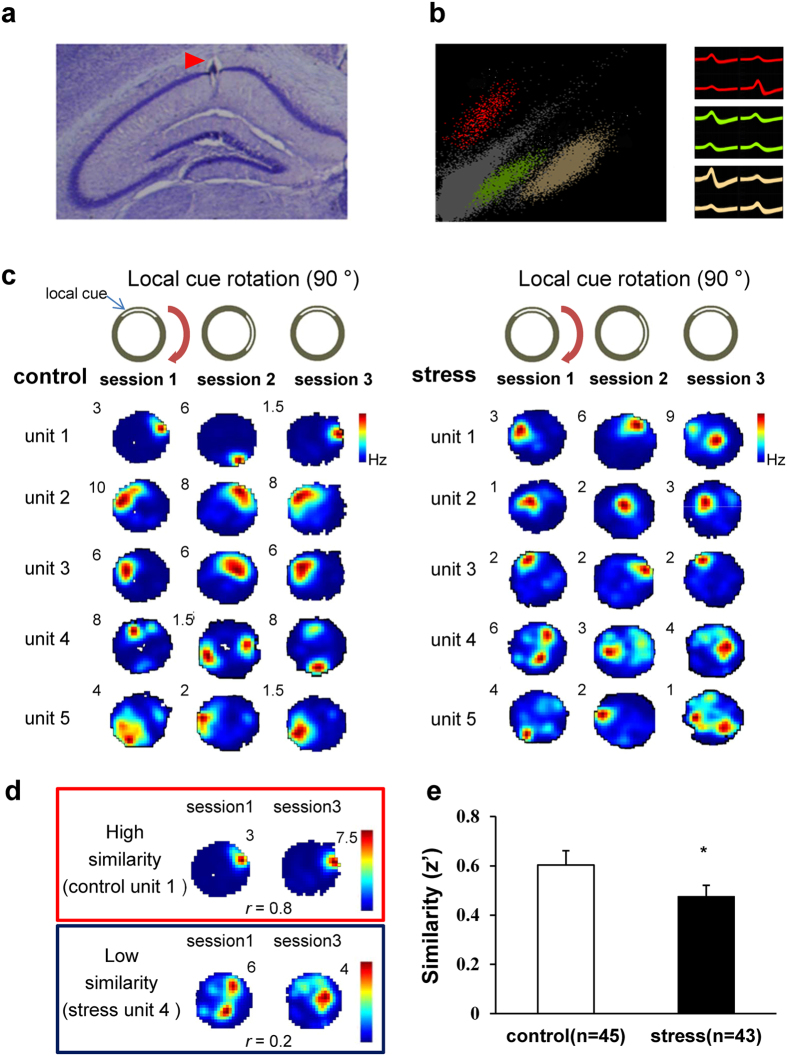
Place cell recording and stability of place cell. (**a**) Photomicrograph example of recording site. (**b**) Example of single unit clusters and corresponding waveforms. (**c**) Place field examples both control and stressed mice (the number on top left of each place map represents peak FR). (**d**) Examples of place fields with high similarity and low similarity between two sessions. (*r* = pixel-by-pixel correlation value between sessions 1 *vs.* 3; a familiar environment). (**e**) Comparison of similarity of place fields between two groups. All values are presented as the mean + SEM (Unpaired two-tailed *t*-test, *P < 0.05).

After 21 days of CRS, dorsal CA1 pyramidal neurons were recorded in mice foraging freely on the recording chamber for three 20-min recording sessions (Fig. 3c). For place cell analysis, we recorded different place cells in the same recording environment by advancing electrodes (10 ~ 20 μm) through the CA1 pyramidal layer each day and then pooled all data. A total of 88 place cells from 6 control mice (n = 45) and 6 stressed mice (n = 43) were included in further analyses. There were no significant group differences on the mean number of cells recorded per mouse (control: 7.5 ± 2.53 cells, stress: 7.16 ± 1.3 cells, *t*_(10)_ = 0.11, *P* = 0.90) and the mean number of recording days (2.5 ± 0.56 days, 3 ± 0.57 days, *t*_(10)_ = −0.62, *P* = 0.54, respectively), suggesting that there were no biases in the unit sample size and the time experienced in the recording environment.

Although there was no visible distinction in place fields (Fig. 3c), quantifications of place cell activities revealed significant group differences in several parameters (Table 1; all data presented as average of 3 recording sessions). The most noticeable change induced by CRS was the reduction of mean FR (*z* = −2.49, *P* = 0.01), In-field firing rate (*z* = −2.52, *P* = 0.01) and Out-field firing rate (*z* = −3.21, *P* < 0.01) compared to control mice. This difference in FRs were unlikely due to motoric and or motivational differences because both control and stressed mice exhibited comparable pellet pursuing speeds (*t*_(31)_ = 0.85, *P* = 0.4). In addition, when the field size was normalized by mean FR to exclude the influence of altered mean FR^34^, there was no significant difference between two groups (*z* = −0.96, *P* = 0.33). Specifically, stressed mice showed lowered spatial coherence (*t*_(86)_ = 1.78, *P* = 0.07) compared to control mice, indicating that the accuracy of prediction towards the peak of place field was lower in stressed mice.

### CRS reduced spatial stability of place fields in a familiar environment

One of the place cell characteristics is to maintain a stable place fields in a familiar environment for long periods of time, which is known to be a neuronal mechanisms underlying behavioral spatial learning and memory^21^,^35^,^36^. Thus, we compared spatial stability of place fields between two sessions for both groups by calculating the pixel-by-pixel cross correlation between 2 place fields (sessions 1 *vs.* 3 with the same cue orientation) (Fig. 3d). This similarity score of stressed mice was significantly lower than that of control mice (*t*_(86)_ = 1.82, *P* = 0.03) (Fig. 3e), suggesting that place cells from stressed mice are less capable of recognizing the same environment following CRS compared to control mice.

### CRS altered cue dependency of place fields

**Figure 4:**
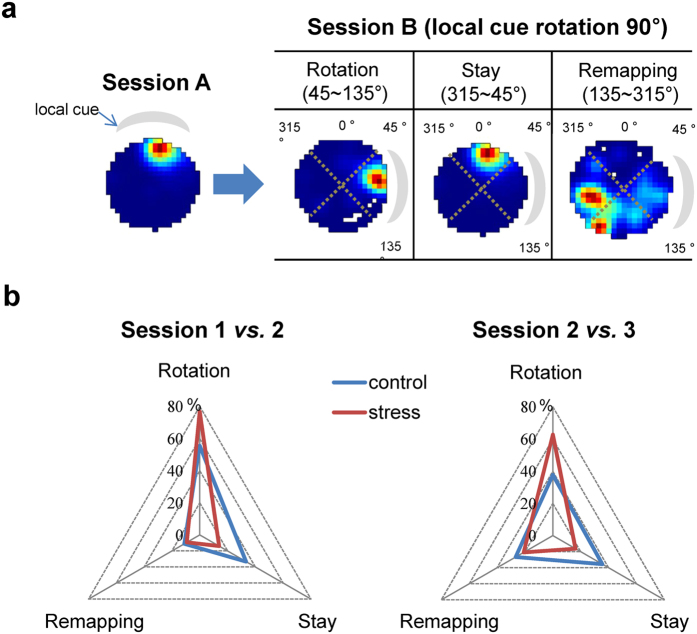
Cue dependency of place field. (**a**) Examples of 3 place field categories that represent the amount of rotation between 2 sessions in the cue rotated environment. (i) “Rotation” category (S-R strategy): place fields rotated within the 90° range (45 ~ 135°) of the new local cue position; (ii) “Stay” category (spatial strategy): place fields stayed within the 90° range (315 ~ 45°) from the original field even when local cue is rotated; and (iii) “Remapping” category: remaining cells with place fields that fit neither “Stay” nor “Rotation” category. (**b**) Classification of place field behavior between control and stressed groups. Plots show proportion (%) of place cells in each category between sessions 1 *vs.* 2 and 2 *vs.* 3. (Chi-square test, *P < 0.05).

The fact that CRS enhanced performance on the visible platform task suggests that stressed mice mainly utilized the local cue of the visible platform more than control mice did (Fig. 2d–f). Hence, the cue dependency of place fields was examined to see if there were any comparable changes with behavioral changes. The place cells were grouped according to the rotation amount of each place field following local cue rotation between sessions 1 *vs*. 2 and 2 *vs.* 3 as “Rotation”, “Stay”, and “Remapping” categories (Fig. 4 and Fig. S6). The amount of place field rotation was obtained by calculating pixel-by-pixel correlation values (similarity index) between two place fields with one place field rotated by every 5° clockwise to find the maximum similarity value (Fig. 4a)^37^.

Two groups showed significant difference in the cue dependency of place field in both comparisons between sessions 1 *vs*. 2 and 2 *vs*. 3 (Chi-square test: *X*^2^ = 13.80, *P* = 0.003 for sessions 1 *vs.* 2; and *X*^2^ = 10.87, *P* < 0.001 for sessions 2 *vs.* 3) (Fig. 4b). In particular, the place fields of stressed place cells showed different tendency of cue-dependency for both “Rotation” as well as “Stay” but not for “Remapping” compared to place fields of control place cells. For example, stressed place cells prefer the salient local cue on cue-rotation (77% (N = 33) for sessions 1 *vs.* 2, and 63% (N = 27) for sessions 2 *vs.* 3) compared to control place cells (56% (N = 25) for sessions 1 *vs.* 2, and 38% (N = 17) for sessions 2 *vs.* 3) while dependency on static distal cues was lower in stress group (12% (N = 6) for sessions 1 *vs.* 2, and 16% (N = 7) for sessions 2 *vs.* 3) than control group (33% (N = 15) for sessions 1 *vs.* 2, and 36% (N = 16) for sessions 2 *vs.* 3).These results suggested the possibilities that CRS prevents place cells from utilizing static distal cues while potentiates the salience of local cues in a changing environment, which is comparable with behavioral changes following CRS.

### CRS altered hippocampal bursting patterns

**Figure 5:**
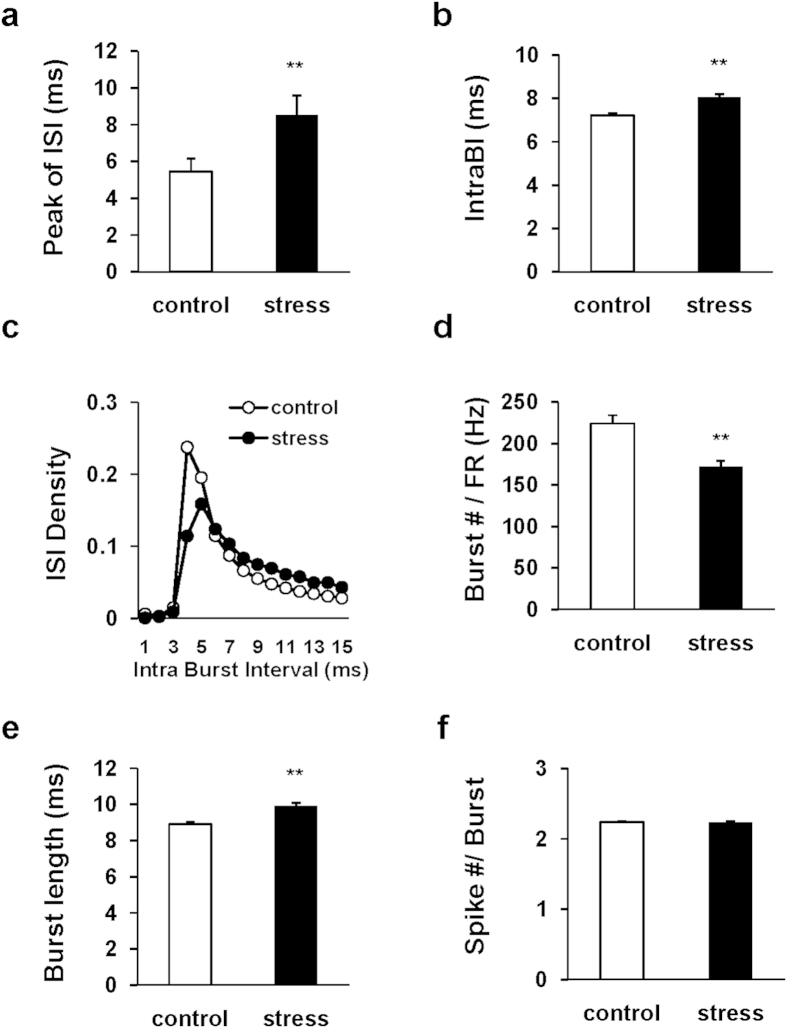
CRS altered bursting pattern. (**a**) Peak time of ISI. (**b**) mean IntraBI (ms). (**c**) Density distribution of the IntraBI between 1^st^ and 2^nd^ spikes of all bursts. (**d**) Burst number (normalized by the mean FR on individual neuron). (**e**) Burst length (ms). (**f**) Spike number within a Burst. All values are presented as the mean ± SEM (Mann-Whitney U test, Unpaired two-tailed *t*-test, *P < 0.05, **P < 0.01).

Interestingly, the place cells from stressed mice showed a significantly prolonged peak time in the inter-spike interval (ISI) histogram (*z* = −3.27, *P* < 0.01) (Fig. 5a). However, when we compared the ISI histogram variability of all spikes for measurement of the distributional dispersion by calculating the coefficients of variations (CV), we found no significant difference between groups (2.64 for control mice *vs*. 2.92 for stressed mice, *z* = −1.22, *P* = 0.22). Since hippocampal pyramidal neurons fire as a complex spike burst, the peak time of ISI histogram of individual place cells mainly represents interval of burst spikes within a burst (IntraBI)^21^. When we analyzed burst spiking, the results revealed that stressed mice showed longer mean IntraBI than control mice (*t*_(86)_ = −2.96, *P* < 0.01) (Fig. 5b). A density distribution analysis on IntraBI also confirmed a significant group difference in the probability distribution of IntraBI between the 1^st^ and 2^nd^ spikes (*X*^2^ = 459, *P* < 0.01) (Fig. 5c). In addition, when the burst # was normalized by mean FR, it was significantly reduced (*t*_(86)_ = 2.88, *P* < 0.01) (Fig. 5d) and burst length (ms) was significantly lengthened in stressed mice (*t*_(86)_ = −3.12, *P* < 0.01) (Fig. 5e), even though the spike number per burst was similar compared to control mice (*t*_(86)_ = −0.02, *P* = 0.98) (Fig. 5f). These results suggest that CRS induces significant temporal alteration of burst spiking patterns as well as the burst frequency.

## Discussion

Exposures to CRS have been found to elevate CORT levels^38^, cause weight loss^39^, reduce dendritic spines in hippocampal pyramidal neurons^28^,^29^, and impair LTP^40^ and spatial learning and memory in rats^12^,^13^,^14^. The present study showed similar endocrine, morphological, physiological and behavioral changes associated with CRS in mice. Furthermore, we extended the effects of CRS on hippocampal functions at the neural computational level by providing novel electrophysiological evidence that CRS decreases the stability of spatial representation, alters the temporal bursting pattern, and enhances the local cue dependency of CA1 place cells.

Previous behavioral studies have shown that while acute stress impaired hippocampal-based learning, it enhanced nonhippocampal-based learning in rats^15^ and humans^41^ in navigation tasks. Administrations of anxiogenic drugs^42^ in rats and CORT in mice^43^ have also been reported to shift learning from hippocampus-based strategy to hippocampal-independent strategy. These findings suggest the possibility that stress enhances the hippocampal-independent learning by virtue of reducing the competing hippocampal-dependent learning. Consistent with this view, a recent human neuroimaging study^16^ showed that stress induced shift in contribution of memory system in probabilistic classification learning (PCL) tasks from hippocampus (a single-cue-based declarative strategy) to striatum (a multicue-based procedural strategy). It appears then stress reduces both navigational^15^,^41^,^42^,^43^ and non-navigational^16^ hippocampal learning irrespective of the types of (e.g., single, multicue, distal, local) cues being processed in the hippocampus. Similar stress effects on navigational learning were observed in the present study, where CRS impaired the performance on a hidden platform (spatial cue) task but enhanced the performance on a visible platform (non-spatial cue) task in mice. However, the neural computation basis for enhanced hippocampal-independent learning has remained unknown.

We found that CRS induced the shift of cue dependency of place cells in response to a local cue rotation, which could be a neuronal substrate underlying CRS induced behavioral changes of a navigational strategy. In general setting of place cell recording, animals are forced to focus on the local cues placed on a high walled recording chamber (over 34 cm height) where no distal cue is visible^44^. However, if both local and distal cues were visible, place cells could follow either a local cue or distal cues. For example, when a salient local cue was rotated while static distal cues were available, some place fields followed the local cue rotation whereas other place fields followed the distal cues^45^. In our low enclosure cylinder (12.7 cm height) recording setup, the place fields of stressed mice showed substantial preference to the local cue (assessed by rotating the cue) compared to control mice. The place fields of stressed mice might thus be strongly controlled by a salient local cue whereas control mice appeared to depend on both local and static distal cues (e.g., location and orientations of camera, food feeder etc.). This indicates that CRS causes spatially-based place fields to switch to local cue-based place fields. As local cue dependent learning appears to be mediated by the prefrontal cortex and the dorsal striatum, structures also implicated in decision-making^46^, reversal learning^32^,^47^, and behavioral flexibility^48^, the increased local cue dependency in the hippocampal place cell activity might also subserve stress effects on other cognitive processes^16^. Although stress increased the local cue-dependency of place fields and altered other physical properties of spiking in place cells, future studies will need to ascertain whether these changes are necessary and accompanied by changes in neural structures mediating non-hippocampal strategy.

In an earlier study, we showed that acute (audiogenic) stress also influenced hippocampal place cells^25^. However, whereas the acute stress effect on place cells was relatively specific to causing instability in FR (but not firing locations), CRS employed in the present study produced broader effects on place cell properties. What, then, may account for CRS effects on the hippocampus at the neural computational level? The present findings can stem from dendritic atrophy^28^,^29^, suppression of neurogenesis^20^, and alterations in synaptic plasticity^40^,^49^ associated with long-term exposures to stress and elevated levels of CORT. Although, CORT administration has been reported to decrease single unit activity in the hippocampus^50^, a recent study found that it did not alter the stability of place fields^51^. Moreover, since place cells were recorded at least 15 hours after the CRS, the present findings are unlikely due to direct influences of CORT. Alternatively, reduced CaMKII protein level following CRS might contribute to altered hippocampal function. A recent finding revealed a close correlation between place field stability and the temporal bursting pattern by showing that αCaMKII mutant mice exhibit disrupted stability of place cells, abnormal bursting patterns as well as impaired spatial learning^34^,^37^,^52^. Since phospho-αCaMKII protein level is a key signaling proteins for LTP in the hippocampus^53^,^54^, CRS-induced reduction of αCaMKII protein level can modify synaptic plasticity as well as spatial stability of place cells.

The CRS-induced changes in the bursting pattern of the CA1 pyramidal cells—via diminishing the burst frequency and prolonging IntraBI—might be the basis for spatial stability in the place fields. The hippocampal bursting pattern has been implicated in synaptic plasticity, such as LTP induced through pairing pre-synaptic activity with post-synaptic bursts in CA1 pyramidal cells^55^,^56^. Furthermore, the complex spike bursting was suggested to play an important role in learning and memory, perhaps by producing a highly sensitive postsynaptic state (i.e., depolarization) necessary for the coinciding pre-synaptic activity to undergo LTP^24^,^57^. Since CRS reduced LTP in the hippocampus^40^,^49^, such change in synaptic plasticity could alter burst properties in stressed mice. Consistent with this possibility is a recent finding demonstrating a close correlation between place field stability and the temporal bursting pattern^37^. Hence, CRS induced temporal alteration of bursting pattern could represent decreased stability of spatial representation of place cells and impaired learning and memory.

Based on the reported association between activity-dependent synaptic plasticity and the cellular mechanism for memory acquisition and consolidation^58^,^59^, abnormal place cell firing pattern may be critical for the retardation of hippocampal-dependent spatial learning and memory following CRS. Clearly, future studies will need to address detailed mechanisms at various levels underlying chronic stress effects on spatial representation and physiological properties of hippocampal neurons.

## Methods and Materials

### Animals

F1 hybrids of C57BL/6 J x 129/SvJae male mice (initially weighing 25 g) were housed individually and maintained on a 12:12 h light/dark cycle (lights on at 8:00 AM) in a climate-controlled vivarium (22 °C). Prior to the experiment, mice were handled daily for 7 days. Daily behavioral and recording procedures took place between 10 AM-1 PM. Both control and stressed mice were placed on a mild food deprivation condition in which one food pellet (3 g) was provided every morning. All experimental procedures were approved and conducted in accordance with the guidelines of the Institutional Animal Care and Use Committee (IACUC) of Korea Institute of Science and Technology (Protocol Number: AP-2009L7020).

### CRS paradigm

The CRS paradigm involved immobilizing the mice using a latex glove and placing them inside their home cage for 6 hrs daily (~11 AM–5 PM during the first 21 days and ~1 PM–7 PM on subsequent days). Because CRS effects were reported to be reversible within 7–10 days^7^, CRS was applied throughout the water maze and place cell recording experiments (Fig. S1).

### fEPSP and synaptic plasticity

In field excitatory postsynaptic potential (fEPSP) measurement, single bipolar metal electrode (FHC, Bowdoinham, ME) was placed at one side of CA1 dendritic region and the glass recording electrode, filled with aCSF, was positioned the other side with 300–500 μm distance. Stimulating current was delivered at 30 second interval from the constant current isostimulator (SC-100, WECO). fEPSP input-output plot was made by applying 100 μsec of currents, from 10 μA to 190 μA to stimulating electrode with 30 μA step-increase. Fiver volley amplitude was used as presynaptic stimulus intensity. Input-output slope was calculated by linear regression method.

For synaptic plasticity, single theta-burst stimulation consists of 10 bursts at 4 Hz, each burst with 5 pulses at 100 Hz, with the same stimulation intensity. Four theta-burst stimulation protocol uses the single theta-burst stimulation four times at 5 minute interval. fEPSP was collected Dagan amplifier (EX-1), filtered at 2 kHz and stored in hard disk of PC. All data acquisition and analysis were done by custom software written in Axobasic 3.1 (Axon Instruments).

### Hidden platform Morris water maze

Animals were trained to find a hidden platform (10 cm diameter, 1 cm under the water surface) placed in a fixed location in a water maze (1.2 m diameter) filled with water (25 °C) made opaque by the addition of nontoxic white paint (Weather tough Forte, Bristol Paints). The water maze was surrounded by a black circular curtain (placed 70 cm away) that held 3 salient visual cues. The releasing point was randomly distributed across 4 quadrants of the pool and the animal was allowed maximum 60 sec to find the hidden platform. If escape did not occur within 60 sec, the animal was manually guided to the platform where they stayed on for 30 sec. The training consisted of 4 trials/day (10 min inter-trial interval, ITI) for 7 days. On training days 4 and 8, animals were given 60 sec probe tests (sans the platform) to test their spatial memory. After 7 days of acquisition, the hidden platform was placed on the opposite quadrant and animals underwent 3 additional days of reversal training and the final probe test.

### Visible platform Morris water maze

Animals were trained to find a visible platform (10 cm diameter, 1 cm above the water surface) marked with a salient black tape for 2 days (4 trials/day, 10 min ITI). If the animal found the platform, the animal remained on the platform for 30 sec. During the retention test, the platform was moved to a new location (adjacent right quadrant). And the animals were released in the pool equidistant from the original and new location^15^. An automated tracking system (Noldus, Netherlands) was used to monitor the animal’s swimming pattern and speed, the number of platform crossing, and the amount of time spent in each of the four quadrants.

### Single unit recording

Mice anesthetized with Zoletil (30 mg/kg, i.p.) were placed on a stereotaxic instrument (David Kopf Instruments, USA) and implanted with a microdrive equipped with four tetrodes slightly above the dorsal hippocampal CA1 region (AP: −1.8 mm, ML: −1.5 mm, DV: −0.6 mm; right hemisphere). A tetrode was made by twisting four strands of polyimide-insulated nichrome microwires (12.5 μm, Kanthal Precision Technology, Sweden) and gently heated to fuse the insulation. Each microwire tip was gold-plated to reduce the impedance to 300–500 kΩ (at 1 kHz). Two types of microdrives were used: a Harlen 4 drive (Neuralynx, Tuscon, AZ) capable of individual manipulations and a custom-made bundle electrode microdrive. Animals were given 7 days of post-operative recovery before commencing the experiment.

The place cell recordings were performed after 21 days of CRS. Tetrodes were gradually advanced (20 μm per day) until complex spike cells were encountered in the CA1 layer. Unit signals were amplified (X 10,000), filtered (600 Hz to 6 kHz), and digitized (30.3 kHz) using the Cheetah data acquisition system (Neuralynx, Tuscon, AZ). The animal’s head position was sampled at 30 Hz by tracking light emitting diodes (LED).

Place cells were recorded in black cylindrical chamber (30 cm diameter, 12.7 cm height) placed on the center of the table surrounded by black curtains in a dimly lit room with white noise (85 dB). Food pellets (20 mg) randomly dropped onto the floor motivated the animal to frequent all areas of the chamber. Within the black cylinder wall, a rectangular white cardboard (26 cm x 12.7 cm) was mounted as a local cue covering 90° arc. The cue arrangement was identical in sessions 1 and 3 whereas the local cue was rotated clockwise 90° in session 2. Three recording sessions (20 min/session) were conducted with 3 min inter-session interval (ITI). The mice were always placed into the center of the chamber in the same direction in the beginning of each recording session and returned to a black box (rectangle, 22 cm × 15 cm) between recording sessions during 3 min.

### Place cell analyses

Single units were isolated using Spike Sort 3D (Neuralynx, USA) (Fig. 3b) and cluster quality was assessed by L-ratio, isolation distance, and inter-spike interval (ISI, >1 ms) in the ISI histogram. Cluster quality was similar between two groups (control: 0.47 ± 0.05, stress: 0.49 ± 0.06, *t*_(86)_ = −0.21, *P* = 0.83, for L-ratio; 17.31 ± 2.88, 15.64 ± 1.29, *t*_(86)_ = 0.52, *P* = 0.59, for isolation distance, respectively). Only place cells which have specific place fields were included in this place cell analyses with a mean FR > 0.2 Hz at least one of three recording sessions. Firing rate maps composed of 1 × 1 cm pixels and smoothed using a 3 × 3 kernel. The pixels with animal’s visit < 1 sec during recording time were excluded from the analyses. The firing map (place field) was represented by FR of each pixel; i.e., the total number of spikes divided by the total time spent in the pixel.

The stability of firing rate map between sessions 1 *vs.* 3 in the same environment was assessed by calculating pixel-by-pixel correlation transformed into Fisher’s Z score for parametric comparisons. The maximum correlation value (rotation degree) for classifying cells as ‘stay’, ‘rotation’ and ‘remapping’ was calculated via pixel-by-pixel correlations of place fields between two sessions with one place field rotated at every 5° from 0° to 360° until maximum correlation value was found (Fig. 4a). Place field size (cm^2^) was defined as the summed area of all pixels that had a higher FR than the mean FR. In-field FR was measured as mean FR within the place field that had a higher FR than mean FR of all pixels while out-field FR was measured as mean FR of pixels that had a lower FR than mean FR of all pixels. Spatial coherence, an index of local smoothness towards the peak of the firing field measuring the dispersion of FR of a place cell in a given environment that shows the pixel to pixel variability of FR, was measured as a pixel-by-pixel correlation between the FR at one pixel and the mean FR of neighboring 8 pixels^37^.

To investigate the properties of the bursting pattern, we defined bursts as events of 2 or more spikes with each spike occurring within 15 ms of its predecessor with progressively decreasing amplitudes^23^. All single unit data were analyzed using customized R-programs^60^.

### Statistical Analysis

All statistical analyses were employed using PASW Statistics (v.18). The two-tailed Student’s t-test was used for statistical analyses of group difference. When variables were not normally distributed, Mann-Whitney U test was used for statistical analyses of CORT levels, dendritic spine numbers, mean firing rate, In-field firing rate, Out-field firing rate, Field size and Peak time of ISI between two groups. One-way repeated measures ANOVA was performed to statistical analyses of the body weight and water maze tests. Chi-square test was used for statistical analyses of group difference in cue dependency and density distribution of IntraBI. Results were reported as mean + SEM and statistical significance was accepted at a *P* values less than 0.05 (*P < 0.05 and **P < 0.01).

## Additional Information

**How to cite this article**: Park, M. *et al.* Chronic Stress Alters Spatial Representation and Bursting Patterns of Place Cells in Behaving Mice. *Sci. Rep.* **5**, 16235; doi: 10.1038/srep16235 (2015).

## Supplementary Material

Supplementary Information

## Figures and Tables

**Table 1:** Effects of CRS on the firing properties of place cells.

Place cell properties	Control	Stress
Mean firing rate, Hz	1.42 ± 0.14	0.91 ± 0.09*
In-field firing rate, Hz	2.43 ± 0.21	1.73 ± 0.13*
Out-field firing rate, Hz	0.23 ± 0.03	0.12 ± 0.01**
Running speed, cm/s	6.98 ± 0.30	6.60 ± 0.32
Field size (cm^2^)/firing rate (Hz)	160.40 ± 20.84	175.49 ± 20.11
Spatial coherence	0.97 ± 0.001	0.96 ± 0.001

Field size (cm^2^) was normalized by mean FR (Hz) of each neuron. All values are presented as the mean ± SEM (Mann-Whitney U test, Unpaired two-tailed *t*-test, *P < 0.05, **P < 0.01).
