# Supplementary material for: Chronic Stress Alters Spatial Representation and Bursting Patterns of Place Cells in Behaving Mice
Source: Sci Rep. 2015 Nov 9;5:16235. doi: 10.1038/srep16235 (PMC4637823; doi:10.1038/srep16235)
Supplement: Supplementary Information [file srep16235-s1.pdf]

Supplementary Information

Chronic Stress Alters Spatial Representation of Place Cells and Bursting Patterns in Mice

Mijeong Park[1,3], Chong-Hyun Kim[1,3], Seonmi Jo[2], Eun Joo Kim[4], Hyewhon Rhim[1,3], C. Justin Lee[2,3], Jeansok J. Kim[4], Jeiwon Cho[1,3*]

[1]Center for Neuroscience [2]Center for Functional Connectomics, Korea Institute of Science and Technology, 5 Hwarang-ro 14-gil, Seongbuk-gu, Seoul 136-791, Korea, [3]Neuroscience Program, Korea University of Science & Technology, Daejeon 305-701, Korea, [4]Department of Psychology, University of Washington, Seattle, WA 98195-1525, USA

*Corresponding author: Jeiwon Cho, Ph.D., Center for Neuroscience, Korea Institute of Science and Technology, 5 Hwarang-ro 14-gil, Seongbuk-gu, Seoul 136-791, Korea; E-mail: jeiwon@kist.re.kr

Supplementary Methods & Materials

Plasma corticosterone hormone levels (CORT). Blood samples were collected on days 1, 10 and 21 via decapitation in the morning to assess the basal corticosterone hormone level [1]. Blood samples from the stressed mice were taken 1 hr after the initiation of the restraint stress in order to measure the peak level of the stress hormone [2]. The blood samples were centrifuged (1000g, 4°C, 10 min), then the plasma samples were separated and stored at -20°C [3]. Plasma corticosterone was analyzed using the Corticosterone Enzyme Immunoassay (EIA) kit (Assay Designs, Ann Arbor, MI) and hormone levels were measured using ELISA reader (Molecular Devices, CA, USA).

Golgi staining. After 21 days of CRS, the stressed mice (N=4) and time-matched control mice (N=4) underwent cervical dislocation/decapitation. The brain was removed and fixed with Golgi staining solution in the manner specified by the FD Rapid GolgiStain Kit (FD NeuroTechnologies, Ellicott City, MD). Coronal sections of 80 μm thickness were cut through the entire hippocampus region using a cryostat (Microm, Germany) at -23°C. Well stained pyramidal neurons were selected and the dendritic spines were counted in 50 μm successive segments of apical dendrites in CA1 and CA3 regions using a microscope (OLYMPUS, BX50, 1000X magnification).

Hippocampal slice preparation and Current-clamp recording. After 21 days of CRS, mice were deeply anesthetized with isoflurane, followed by decapitation. Dorsal hippocampal slices (300 μm thickness) were prepared from each side of one

mouse brain. Hippocampal containing region was dissected out using cold oxygenated (95%O2/5%CO2) artificial CSF(aCSF) (mM): NaCl 124, KCl 2.5, $NaHCO_3$ 26, $NaH_2PO_4H_2O$ 1.25, Glucose 11, $CaCl_2H_2O$ 0.5, $MgCl_2$ 5, pH 7.3. Slices were kept on the surface of cell culture inserts in an incubation chamber to which humidified oxygen was continuously supplied for storage, and at least one hour after dissection, one or two slices were transferred to the recording chamber for the recording. The recording aCSF has 2.5mM Ca and 1.3mM. For whole cell recording, pyramidal neurons were selected in CA1 pyramidal cell body layer by infrared differential interference contrast (IR-DIC) microscopy (Olympus BX51W1) with a 40x objective. The intracellular solution has the following components (mM): K-gluconate 120, KCl 15, $MgCl_2$4, HEPES 10, MgATP4, Na3GTP 0.3, EGTA 0.1, Phosphocreatine 7, pH 7.4 (about 300 mOsm). The traces were filtered at 2 kHz.

Histology. Upon the completion of recordings, the mice were overdosed with 2% Avertin and a small current (10-30 μA, 10 sec) was passed through one of 4 wires in the tetrode to mark the recording site. The mice were transcardially perfused with 10% formalin and then brains were extracted and fixed further in 10% formalin at room temperature. Coronal sections (50 μm) were cut through the entire hippocampus region using a cryostat (Microm, Germany) at -23°C. Brain slices were mounted on the slides and then stained with Cresyl Violet (Fig. 3a).

Western Blotting. After 21 days of CRS, mice were anesthetized by i.p. injection of tribromoethanol (Avertin, 20 mg/mL). The brain was quickly excised from the skull and submerged in ice-cold PBS. After cooling, CA1 region of the hippocampus from

each mouse were separated and stored at -70°C until analysis. Each sample was homogenized and lysed with RIPA buffer 20g of proteins (as estimated using the BCA reagent; Pierce) were separated by SDS-PAGE using 10% polyacrylamide gels and blotted onto PVDF membranes. The blots were incubated overnight at 4°C with either rabbit anti-CAMK2 (phospho T286) antibody (1:1000, Abcam), mouse anti-CAMK2 antibody (1:1000, Abcam). For protein loading control, each blot was incubated with mouse anti-GAPDH antibody (1:1000, Abcam). Blots were then washed and incubated with horseradish peroxidase-conjugated goat anti-mouse, followed by washing and detection of immunoreactivity with enhanced chemiluminescence (Amersham Biosciences). The band intensity was acquired and analyzed by Image Quant LAS4000 (General Electric Company).

Reference

1. Seasholtz, A.F. & Rozeboom, A.M. Mineralocorticoid receptor overexpression in forebrain decreases anxiety-like behavior and alters the stress response in mice. *Proceedings of the National Academy of Sciences of the United States of America* **104**, 4688-4693 (2007).
2. Magarinos, A.M. & McEwen, B.S. Stress-induced atrophy of apical dendrites of hippocampal CA3c neurons: involvement of glucocorticoid secretion and excitatory amino acid receptors. *Neuroscience* **69**, 89-98 (1995).
3. Pavlides, C., Nivon, L.G. & McEwen, B.S. Effects of chronic stress on hippocampal long-term potentiation. *Hippocampus* **12**, 245-257 (2002).

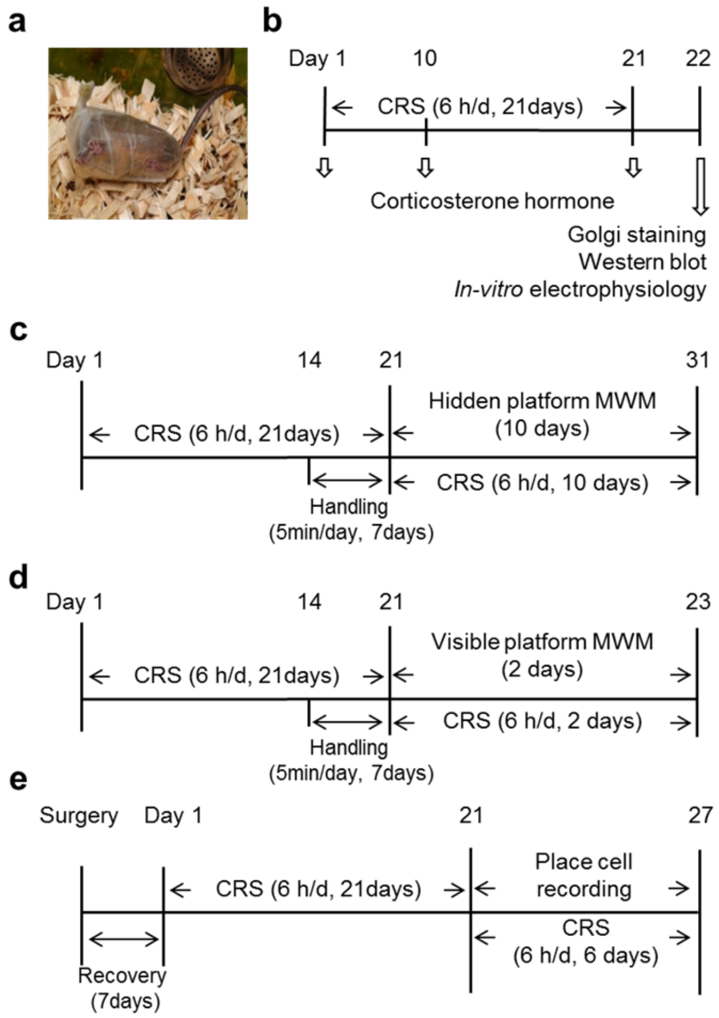

Figure S1. Experimental designs. (a) Photograph of the restraint stress. Diagram of the experimental designs for **(b)** corticosterone hormone measurement, Golgi staining, western blot, *In vitro* electrophysiology, **(c)** hidden platform Morris water maze (MWM) test, **(d)** visible platform MWM, and **(e)** the place cell recording experiment in freely moving mice.

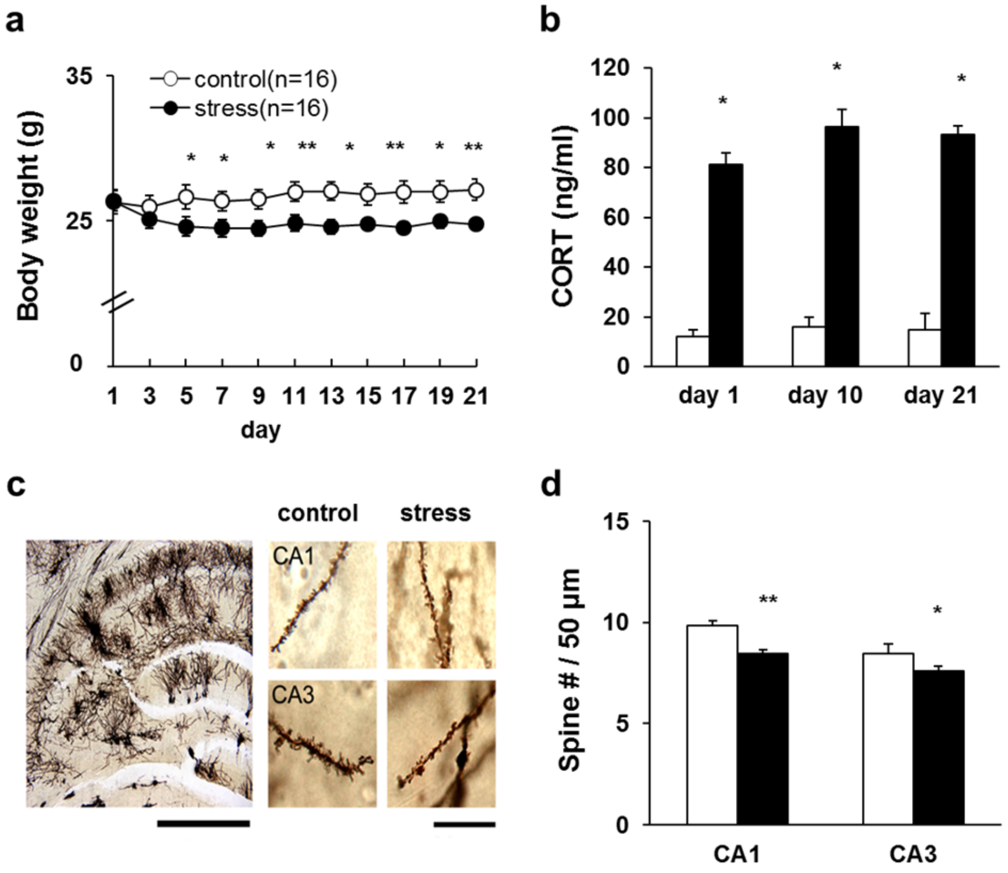

Figure S2. General effects of CRS. (a) Body weight across 21 days. **(b)** CORT levels from three sampling time points. **(c)** Photomicrograph of transverse hippocampal section with Golgi staining (100X); Apical dendritic branches of CA1 and CA3 pyramidal neurons (1000X). Scale bar = 50 µm. **(d)** Dendritic spine numbers from CA1 (N=14 cells from 4 control mice and N=13 cells from 4 stressed mice) and CA3 (N=18 cells from 4 control mice and N=14 cells from 4 stressed mice) pyramidal neurons. All values are presented as the mean$\pm$SEM (One-way repeated ANOVA, Unpaired two-tailed *t*-test, Mann-Whitney U test, *$P < 0.05$, **$P < 0.01$).

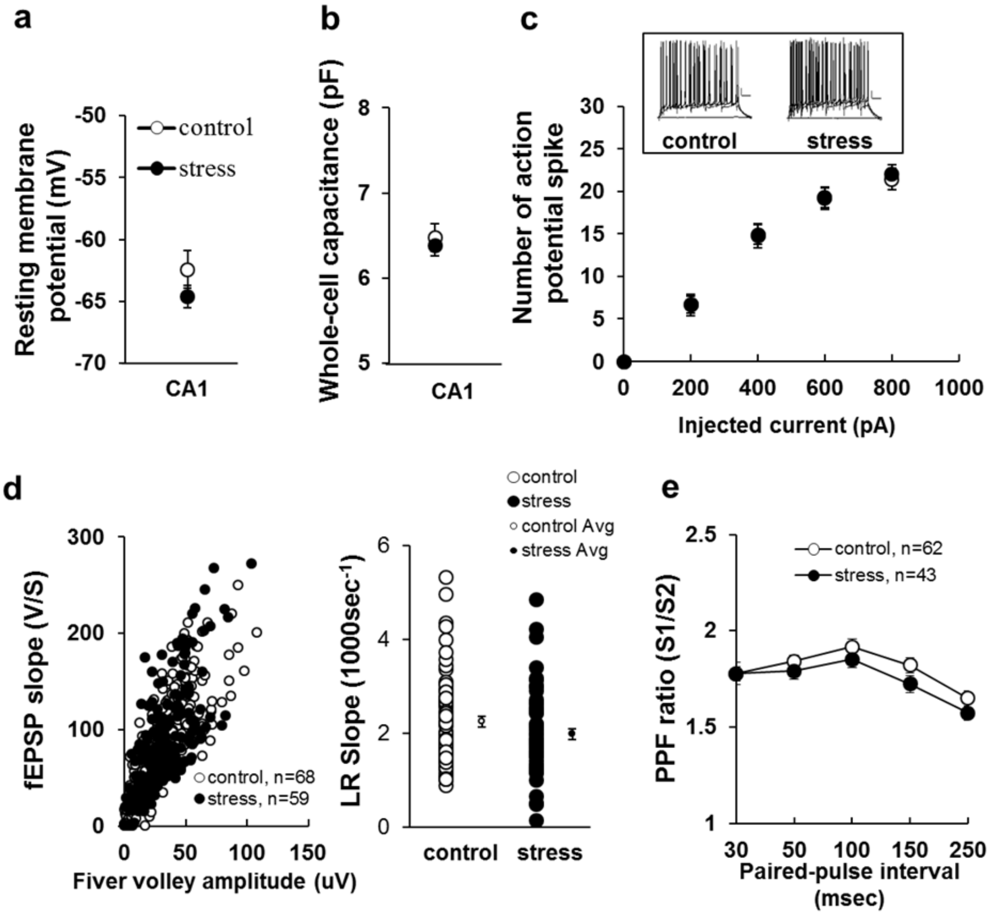

Figure S3. Action potential firing properties induced by current injection of hippocampal CA1 pyramidal neuron. (a) Resting membrane potential (mV) of dorsal CA1 neurons (*P* = 0.46). **(b)** Whole-cell membrane capacitance (pF) of dorsal CA1 neurons (*P* = 0.46). **(c)** Number of action potential firings induced by 250 msec depolarizing current injection into dorsal CA1 neurons, starting from 0 to 800 pA by 200 pA steps. Inlet traces are the example traces overlapped made at five different current injections. All samples were collected from dorsal CA1 neurons (n=14) in control mice and dorsal CA1 neurons (n=17) in stressed mice (*P's* > 0.9). (**d**) Input-Output relationships of basal evoked synaptic transmission. Left, field EPSP slopes in response to six-step incremental stimulation current strengths. Right, linear

regression value of slopes from each slice of the left I-O plot (*P* = 0.11). (**e**) Paired-pulse ratio of fEPSP responses. The ratio of the 2nd fEPSP slope over the 1st fEPSP slope was measured at CA3 to CA1 synapses in hippocampal slices (*P's* > 0.1). All values are presented as the mean±SEM (Unpaired two-tailed *t*-test).

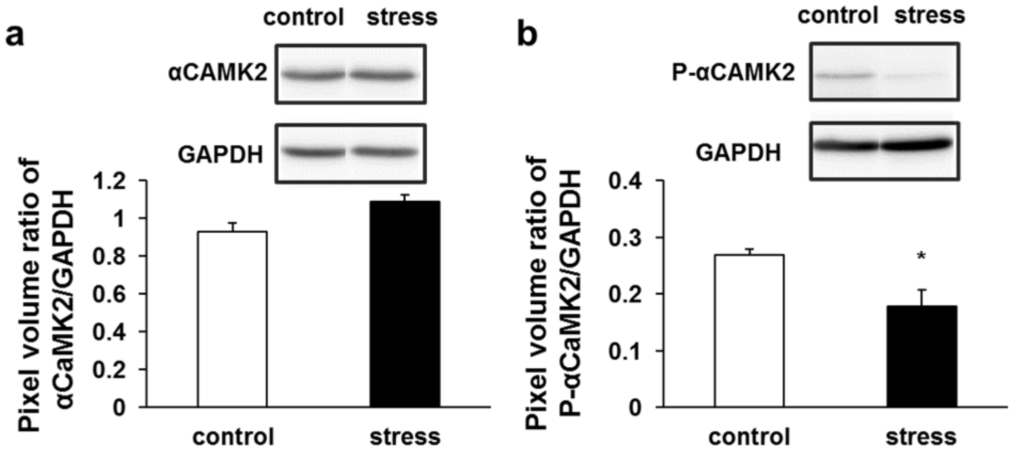

Figure S4. Western blot analysis of the effect of CRS on the αCaMK2 and phospo-αCaMK2 in CA1 hippocampal region. Representative immunoblotting bands and pixel volume ratio of **(a)** αCaMK2/GAPDH and **(b)** phospo-αCaMK2/GAPDH of CA1 hippocampal region from control (n=5) and stressed (n=6) mice. All values are presented as the mean±SEM (Unpaired two-tailed *t*-test, $^*P < 0.05$).

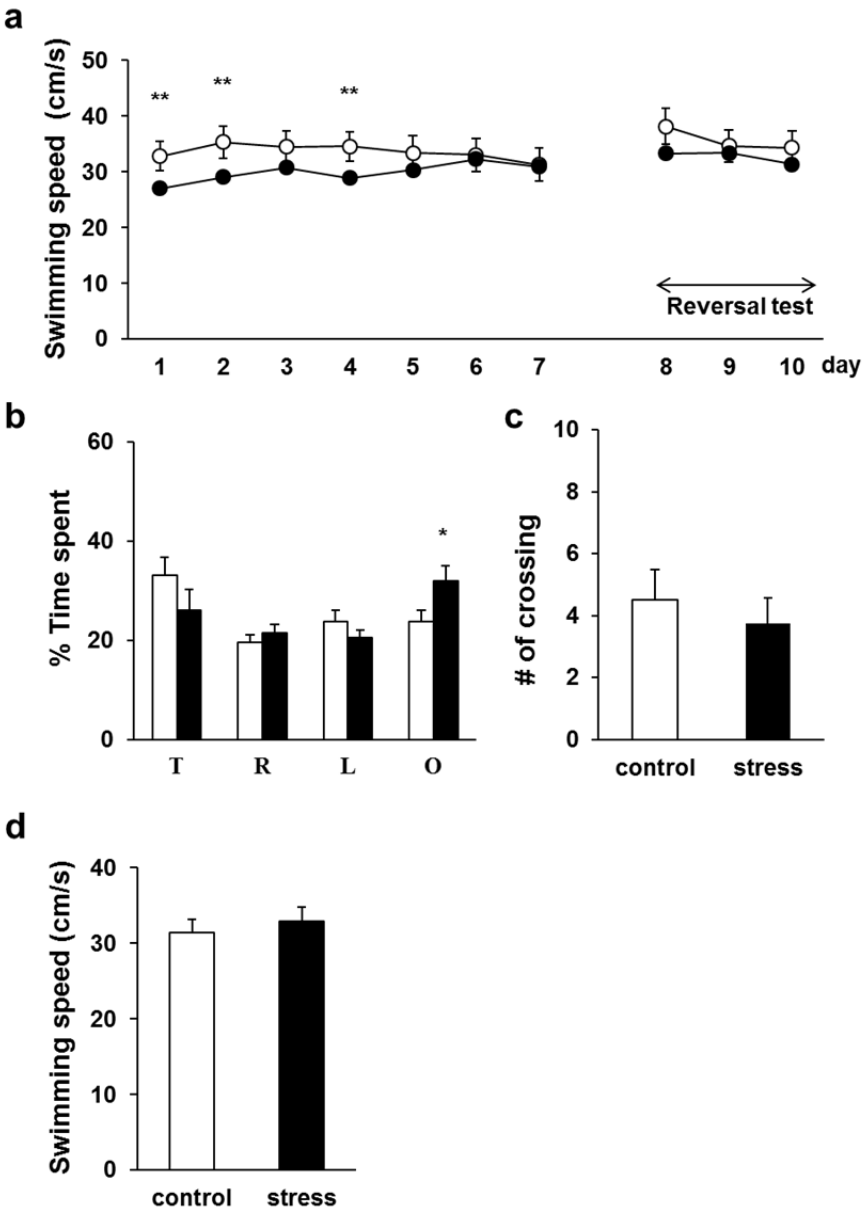

Figure S5. Hidden platform and visible platform Morris water maze tasks. (a) Swimming speed during acquisition and reversal learning periods. Two groups showed significant difference in the swim speed during acquisition period (day1, $t_{(132)} = 6.45$, $P < 0.01$; day2, $t_{(132)} = 6.29$, $P < 0.0$; day4, $t_{(132)} = 5.37$, $P < 0.01$). **(b,c)** Reversal probe tests **(b)** The percent time spent swimming in 4 quadrants. The stressed mice spent more time searching for the opposite quadrant (acquisition

target quadrant) than control mice ($F_{(1,31)}$ = 0.16, P = 0.05, main effect of group; $F_{(1,93)}$ = 2.1, P = 0.098, main effect of group x day interaction; $t_{(31)}$ = -2.18, P = 0.046, in the opposite quadrant). T, target quadrant (reversal target quadrant); R, right quadrant; L, left quadrant; O, opposite quadrant (acquisition target quadrant). **(c)** The platform crossing number ($t_{(31)}$ = 0.64, P = 0.52). **(d)** Swimming speed during the retention test (trial 9) in the visible platform test (see Fig 2d). All values are presented as the mean$\pm$SEM (One-way repeated ANOVA, Unpaired two-tailed *t*-test, *$P < 0.05$, **$P < 0.01$).

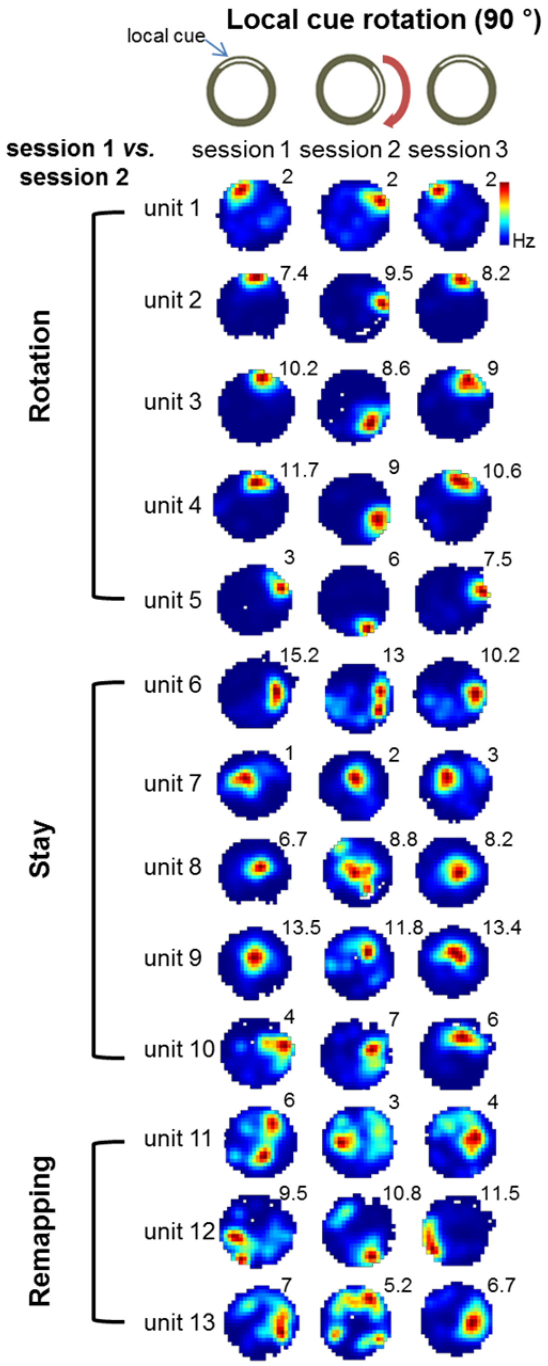

Figure S6. Examples of cue dependency between session 1 and session2 during 3 recording sessions. In session 2 (local cue rotation), unit # 1 to 5 (Rotation) rotated their place fields to follow the local cue rotation and unit # 6 to 10 (Stay) maintained their place fields in the same position as session 2. The place fields of unit # 11 to 13 (Remapping) showed remapping between session 1 and 2.

When place fields were compared between session 1 and 3, most units showed similar place fields. The number on top right of each place map represents peak FR.
